# Chronic disease clusters are associated with prolonged, bothersome, and multisite musculoskeletal pain: a population-based study on Northern Finns

**DOI:** 10.1080/07853890.2023.2177723

**Published:** 2023-02-11

**Authors:** Eveliina Heikkala, Petteri Oura, Markus Paananen, Emma Ho, Paulo Ferreira, Christophe Tanguay-Sabourin, Jaro Karppinen

**Affiliations:** a Research Unit of Population Health, University of Oulu, Oulu, Finland; b Medical Research Center Oulu, University of Oulu and Oulu University Hospital, Oulu, Finland; c Rovaniemi Health Center, Rovaniemi, Finland; d Western Uusimaa Wellbeing Services County, Social and Health Care Services, Espoo, Finland; e Sydney Musculoskeletal Health, School of Health Sciences, Charles Perkins Centre, University of Sydney, Sydney, Australia; f Sydney Musculoskeletal Health, The Kolling Institute, School of Health Sciences, University of Sydney, Sydney, Australia; g Alan Edwards Pain Centre for Research on Pain, McGill University, Montreal, Canada; h Faculty of Medicine, Université de Montréal, Montreal, Canada; i Rehabilitation Services of South Karelia Social and Health Care District, Lappeenranta, Finland

**Keywords:** Musculoskeletal pain, pain severity, chronic diseases, cohort study

## Abstract

**Background:**

Chronic diseases often accumulate with musculoskeletal (MSK) pain. However, less evidence is available on idiosyncratic patterns of chronic diseases and their relationships with the severity of MSK pain in general MSK pain populations.

**Material and methods:**

Questionnaire-based data on physician-diagnosed chronic diseases, MSK pain and its dimensions (frequency, intensity, bothersomeness, and the number of pain sites), and confounders were collected from the Northern Finland Birth Cohort 1966 at the age of 46. Latent Class Analysis (LCA) was used to identify chronic disease clusters among individuals who reported any MSK pain within the previous year (*n* = 6105). The associations between chronic disease clusters, pain dimensions, and severe MSK pain, which was defined as prolonged (over 30 d within the preceding year), bothersome (Numerical Rating Scale >5), and multisite (two or more pain sites) pain, were analyzed using logistic regression and general linear regression models, adjusted for sex and educational level (n for the full sample = 4768).

**Results:**

LCA resulted in three clusters: *Metabolic* (10.8% of the full sample), *Psychiatric* (2.9%), and *Relatively Healthy* (86.3%). Compared to the *Relatively Healthy* cluster, the *Metabolic* and *Psychiatric* clusters had higher odds for daily pain and higher mean pain intensity, bothersomeness, and the number of pain sites. Similarly, the odds for severe MSK pain were up to 75% (95% confidence interval: 44%–113%) and 155% (81%–259%) higher in the *Metabolic* and *Psychiatric* clusters, respectively, after adjustments for sex and educational level.

**Conclusions:**

Distinct patterns of chronic disease accumulation can be identified in the general MSK pain population. It seems that mental and metabolic health are at interplay with severe MSK pain. These findings suggest a potential need to screen for psychiatric and metabolic entities of health when treating working-aged people with MSK pain.

**Key messages**
- This large study on middle-aged people with musculoskeletal pain aimed to examine the idiosyncratic patterns of chronic diseases and their relationships with the severity of musculoskeletal pain. Latent class cluster analysis identified three chronic disease clusters: *Psychiatric*, *Metabolic*, and *Relatively Healthy*. People with accumulated mental (*Psychiatric* cluster) or metabolic diseases (*Metabolic* cluster) experienced more severe pain than people who were relatively healthy (*Relatively Healthy* cluster). These findings suggest a potential need to screen for psychiatric and metabolic entities of health when treating working-aged people with MSK pain.

## Introduction

Multimorbidity, i.e. being affected by two or more chronic diseases, is an ongoing health challenge worldwide [1]. While musculoskeletal (MSK) pain often acts as a key element in the multimorbidity patterns in the general population [2,3], multimorbidity itself is also a substantially prevalent and consequential phenomenon in pain populations [4–6]. Recent studies have shown that compared to individuals with MSK pain only, people with MSK pain and multimorbidity tend to experience more intense, bothersome, or widespread pain [4,6–10], and poorer outcomes in terms of health-related quality of life, physical functioning, and work ability [6,11,12]. Given the well-established burden of MSK pain at both individual- and societal levels [13,14], these findings are worrisome due to aging populations and the increasing prevalence of chronic diseases and multimorbidity in particular [15].

However, not all individuals with chronic diseases and MSK pain suffer from severe, disabling pain [6]. Hence, identification of individuals with the most unfavorable profile of chronic diseases in relation to the severity of MSK pain (and thus clinically relevant pain) is of importance to provide evidence for trials on MSK pain interventions and management and potentially characterize those who are regarded as ‘high-users of healthcare services’ [16]. To date, prior pain population studies aiming to disentangle the association between multimorbidity and severity of MSK pain have mostly relied on counting the number of chronic diseases or focused only on single diseases [4–10,17], thus not fully meeting this goal as different patterns of chronic diseases may possess divergent roles in pain severity/related disability [18,19]. The few studies using other approaches have been conducted in specific MSK populations, e.g. among patients with osteoarthritis, low back pain, or rheumatic diseases [19–21]. As MSK pain is often non-specific by nature [22] – that is, there are no specific pathological causes – a wider approach accounting for all types of MSK pain would increase clinical applicability. Altogether, there are no studies investigating the association between distinct patterns of chronic disease accumulation and MSK pain severity where data have been drawn from a general pain population.

Therefore, this study had several aims. Firstly, we aimed to identify clusters of chronic diseases among middle-aged individuals who reported any MSK pain in the previous year. This was conducted by means of latent class analysis (LCA), which is a widely utilized method for studying the accumulation of several factors with complex interrelationships [23]. Secondly, we aimed to study whether the identified clusters were differently associated with MSK pain dimensions, including frequency, intensity, bothersomeness, and number of pain sites. Finally, we aimed to identify the cluster(s) whose participants had the highest odds of suffering from prolonged, bothersome, and multisite MSK pain (‘severe MSK pain’). Our hypothesis was that at least one of the identified chronic disease clusters would be associated with worse pain outcomes, determined by higher odds of daily pain and a higher level of pain intensity, bothersomeness and a higher number of pain sites, and finally, with a higher odds of severe MSK pain, compared to the healthiest cluster.

## Material and methods

### Study sample

Members of the Northern Finland Birth Cohort 1966 (NFBC1966) comprised the population of the study [24]. They are children of mothers who lived in Oulu or Lapland (the Northernmost provinces of Finland) in 1966 and whose expected date of delivery was between 1st Jan and 31st Dec 1966 (*n* = 12,231; 96% of all births in the area). At the age of 46 years, participants who were alive and whose addresses were known were contacted and invited to complete four postal questionnaires (*n* = 10,331 [25]). A total of 7146 individuals (69% of the target population) took part in the data collection. The study was approved by the Ethics committee of the Northern Finland Hospital District (94/2011, 12.12.2011) and followed the Declaration of Helsinki. The permission to handle pseudonymized data was given by the NFBC Project Center who administer the NFBC1966 data use and storage.

### Chronic diseases

The selection of chronic diseases was based on their non-traumatic nature and on the consideration that they were not primarily pain-inducing. In addition to obesity (body mass index 30 kg/m^2^ or over, calculated from self-reported weight and height [26]), which was also considered as a chronic disease [27], the chronic diseases investigated in this study were: respiratory disease (asthma or bronchial dilatation/chronic bronchitis), hypertension, heart failure, ischemic heart disease, diabetes (type 1 and 2 were united), thyroid disease (either hypothyroidism or hyperthyroidism), celiac disease, inflammatory bowel disease, psoriasis, epilepsy, stroke or other neurological disease, mental health disorder (psychosis, depression, or other mental health disease), substance use disorder (alcohol disorder or other substance use disorder), and sleep apnea. Participants were asked whether they had any of these 14 chronic diseases diagnosed by a medical physician and were required to select ‘yes’ or ‘no’ for each disease. Those with missing answers were considered as not having the corresponding disease. Individuals who did not provide any responses to the chronic disease questionnaire were excluded from the study, as stated above.

### Musculoskeletal (MSK) pain

In the questionnaire, participants reported MSK pain and its frequency in eight body parts (neck, shoulder, arms/elbows, wrists/hands, lower back, hips, knees, and ankles/feet) by selecting whether they had no pain, or had pain on 1–7 d, on 8–30 d, on more than 30 d but not daily, or daily within the last 12 months [28]. Those who reported no pain at each location were excluded, as stated above. The highest frequency of any of these MSK locations was regarded as the overall frequency of MSK for each participant. This frequency was then divided into four categories: (1) on 1–7 d, (2) on 8–30 d, (3) on more than 30 d but not daily (‘>30 d’), and (4) daily. To increase power, the first two categories were combined as ‘≤30 d’.

Participants who reported any MSK pain were asked to estimate their overall pain intensity and overall pain bothersomeness on a Numerical Rating Scale (NRS) of 0–10. In the NRS, 0 equaled ‘no pain’ and 10 equaled ‘extremely intense pain/total disability’. Inquiry of bothersomeness included three different NRS scales (0–10) which assessed disability at work, during leisure time, and during sleep. The highest reported NRS value for any of these scales was considered as the level of pain bothersomeness. The number of pain sites was defined as the sum of the reported pain locations (from 1 to 8 potential locations). All pain dimension variables, apart from frequency, were continuous variables [28].

Finally, we evaluated the severe MSK pain profile using the following dichotomous pain variable: yes (prolonged [over 30 d], bothersome [NRS >5; [29]], and multisite [two or more pain sites] pain), or no (did not fulfill the criteria of the ‘yes’ category). The latter category was used as the reference.

### Confounders

We considered sex [22,30] and educational level (a proxy for socioeconomic status) [30,31] as confounders. The highest education level until the age of 46 years, which was asked in the questionnaire as follows: ‘What is your basic education?’ and ‘What is your vocational education?’ was divided into three categories: (1) compulsory or no basic education, (2) secondary (upper secondary or vocational school), and (3) tertiary (university or university of applied sciences) [32].

### Data analysis

**Figure 1.**
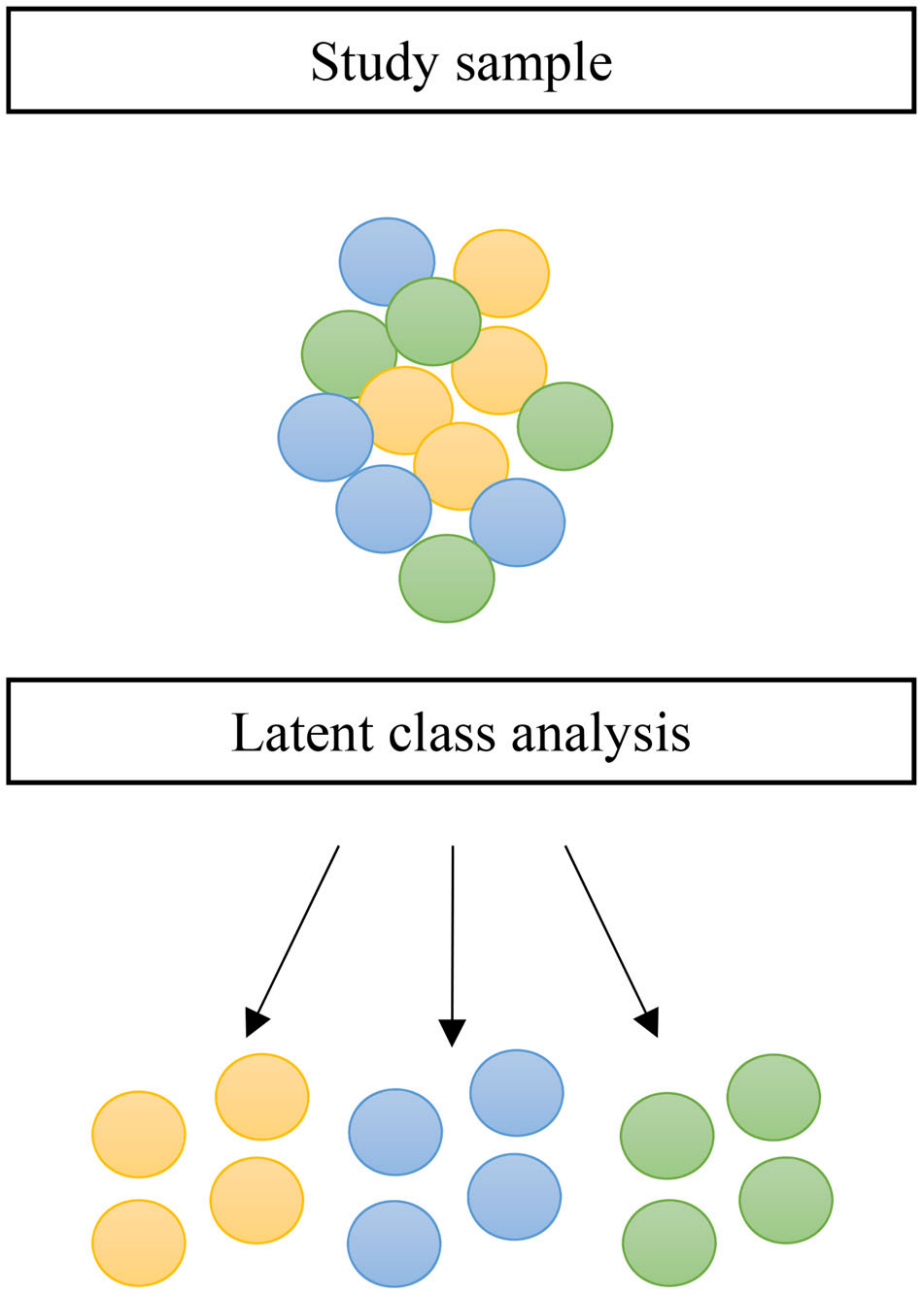
Schematic modelling of latent class analysis.

LCA was utilized to study the accumulation of chronic diseases in this population. LCA is a statistical method designed to divide heterogeneous populations into homogeneous groups (‘clusters’) in terms of studied variables (Figure 1). LCA assumes that there exists an uncovered, latent variable which explains the co-occurrence of chronic diseases within each cluster and classifies individuals into the most probable cluster, based on the posterior cluster membership probabilities. The fundamental idea is that participants in a certain cluster are more similar to each other than to participants in other clusters. In relation to other clustering-related analytic methods, the main advantages of LCA include a probability-based approach and formal goodness-of-fit indices [33,34].

Goodness-of-fit indices help to identify the cluster model that describes the study sample the best from models with a varying number of clusters. We used the following fit indices to evaluate the models: the Bayesian information criterion (BIC), entropy, and bootstrapped likelihood ratio test (BLRT). With respect to BIC, lower values indicate higher model fit, while a higher entropy (range 0-1) suggests a greater accuracy in the classification of the participants into the clusters [35,36]. In the BLRT statistics, the neighboring cluster models are compared, with a low *p*-value stressing the superiority of the qualified model to the model with one less cluster [36].

LCA was performed for participants who reported any MSK pain within the previous year and who had chronic disease data available (*n* = 6105). All 15 chronic diseases were entered into the LCA as dummy variables. The number of models tested was limited to six as the sizes of some of the clusters were small in the five- and six-cluster models (≤1%) (Table 1). Moreover, these models were not superior to more parsimonious solutions in respect of goodness-of-fit statistics. The prevalence of each chronic disease within the clusters was calculated to characterize identified clusters and the statistical significance of the differences in the prevalence rates were studied using the chi-square test (*p*-value < .05 was considered as a liberal and *p*-value < .001 as a conservative statistical threshold after Bonferroni correction to account for multiple comparisons).

**Table 1.** Goodness-of-fit statistics for a one-cluster model through to a six-cluster model.

Number of clusters	Relative sizes of the clusters	APMP	Log-likelihood	BIC	Entropy	BLRT*
One	1.00	1.00	−18,570.068	37,270.890	–	–
Two	0.14/0.86	0.79/0.92	−18,098.963	36,468.149	0.642	<0.001
**Three**	**0.03/0.11/0.86**	**0.77/0.77/0.93**	−**17,985.675**	**36,381.042**	**0.774**	**<0.001**
Four	0.03/0.01/0.11/0.86	0.77/0.81/0.79/0.89	−17,938.788	36,426.739	0.755	<0.001
Five	<0.01/0.05/0.01/0.78/0.15	0.90/0.68/0.70/0.81/0.72	−17,919.226	36,527.085	0.694	0.020
Six	<0.01/0.06/0.08/0.01/0.08/0.78	0.87/0.68/0.67/0.75/0.64/0.75	−17,903.775	36,635.652	0.620	1.000

APMP: Average posterior membership probability; BIC: Bayesian information criterion; BLRT: Bootstrapped likelihood ratio test. Lower BIC values and higher entropy indicate higher model-fit.

* A low *p* value stresses the superiority of the qualified model to model with one less cluster.

The selected model is bolded.

In addition, we tested the statistical significance of the differences in the distribution of categorical and continuous background and pain variables between chronic disease clusters by means of the chi-square test and Kruskal–Wallis test, respectively. Prevalence of single pain locations and concurrent MSK pain locations (e.g. lower back pain and neck pain or knee pain and hip pain) within each cluster were also examined, and prevalence differences of single pain locations between clusters were tested by chi-square test. In these analyses, *p* value < .05 was considered as statistically significant. The effect estimates (odds ratios [ORs] and beta [β] coefficients) were obtained from logistic regression (binomial and multinomial) and general linear regression models, with 95% confidence intervals (CIs). β indicates the mean difference of the continuous outcome variable between studied groups. For each outcome, their own model was constructed, presented as unadjusted and adjusted for sex and educational level. Both these confounders were associated with both the exposure (chronic disease clusters) and the outcomes (pain dimensions and severe MSK pain) in the logistic models (*p* < .05 for all), supporting their inclusion in the final analyses. SPSS Version 27.0 (IBM, Armonk, NY, USA) and Mplus version 8.4 (Muthén and Muthén, Los Angeles, CA, USA) were used for the analyses.

## Results

### Study sample

Of the 7146 individuals who participated in the 46-year data collection of the NFBC1966, 53 individuals declined participation, 606 had missing data on chronic diseases and MSK pain, and 382 reported no MSK within the preceding year. Of the remaining 6105 participants, the full data were available for 4768 (46% of the initial target population).

### Selection of the final LCA model

Across the LCA model candidates, a three-cluster model presented the lowest BIC value and the highest entropy (Table 1). *p* value provided by BLRT statistics was also low (<.001). Therefore, the three-cluster solution was considered to fit the data best and selected as the final model.

### Characteristics of the latent class clusters

**Figure 2.**
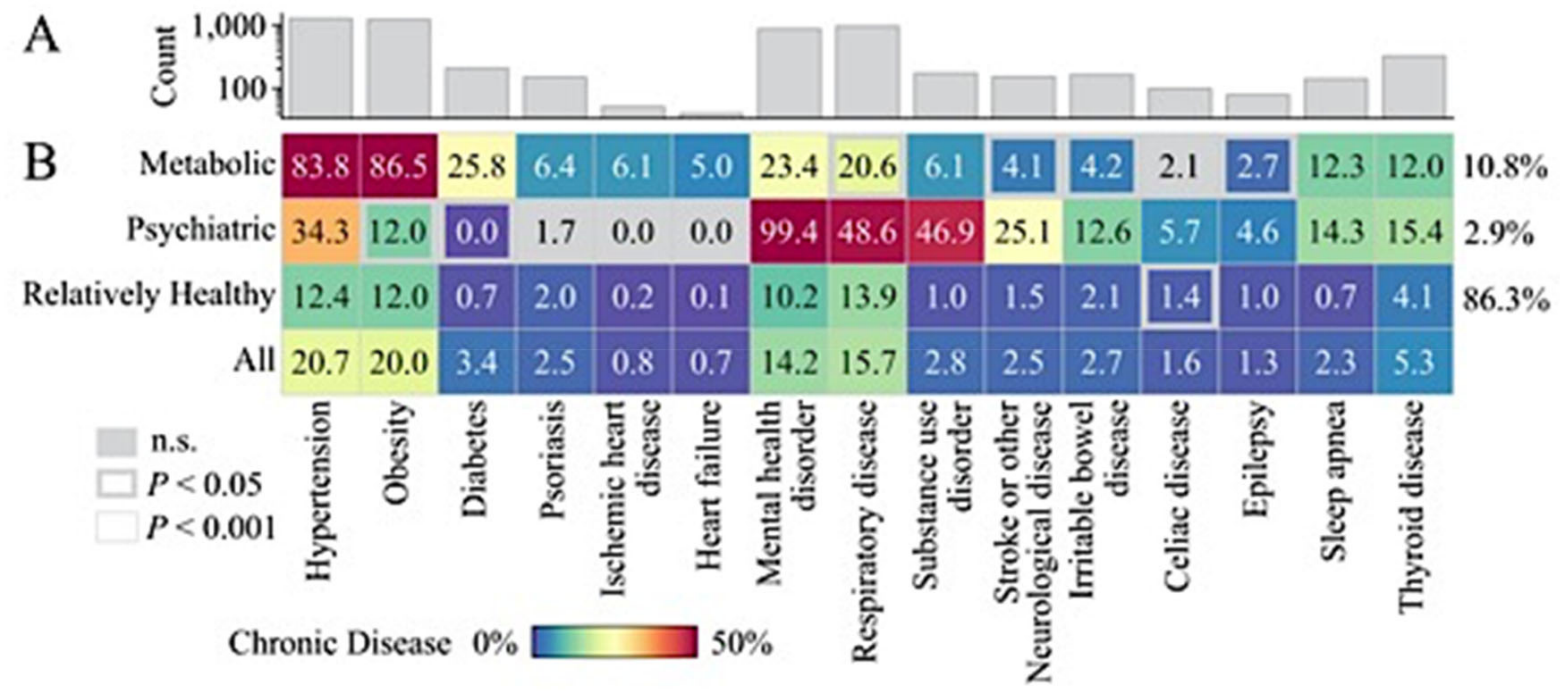
Characteristics of the latent class clusters (*n* = 6105). (A) Count of participants (log-scaled for visualization) in the 15 studied chronic diseases. (B) Prevalence of chronic diseases within clusters and within all participants combined (all participants for reference only). Group comparisons were tested using chi-squared tests for independence to examine chronic diseases significantly represented across each cluster (one cluster vs. all-else). A liberal (*p* < .05) and conservative (*p* < .001) statistical thresholds were examined (for reference: Bonferroni correction < 0.0033). Cluster prevalence is shown in right-hand side of the matrix. n.s.: non-significant.

The three clusters obtained from LCA of 6,105 participants were identified as a ‘*Psychiatric*’ cluster, a ‘*Metabolic*’ cluster and a ‘*Relatively Healthy*’ cluster (Figure 2). The *Psychiatric* cluster (Cluster 1, prevalence within the full sample: 2.9%), comprised participants who virtually all had a mental health disorder (99.4%). In addition, nearly half of them had a substance use disorder (46.9%) and a respiratory disease (48.6%). The presence of stroke or other neurological diseases (other than epilepsy) was also relatively common in this cluster (25.1%). The *Metabolic* cluster (Cluster 2, 10.8% of the full sample) included participants who were most likely to be obese (86.5%), have hypertension (83.8%) and diabetes (25.8%) (*p* < .001). The prevalence rates of heart failure (5.0%) and ischemic heart disease (6.1%) were also higher in this cluster, compared to other clusters (*p* < .001). The *Relatively Healthy* cluster (Cluster 3, 86.3% of the full sample), were least likely to have any chronic disease; the prevalence of each chronic disease was lower than in the full sample (*p* < .001 for all chronic diseases).

Background and pain variables were also compared across the three clusters (Table 2, *n* = 4768). The *Metabolic* cluster had a slightly higher percentage of men compared to the other clusters (51.9% vs. 49.3% and 43.7% in the *Psychiatric* and *Relatively Healthy* clusters, respectively, *p* = .004), while the *Psychiatric* cluster presented a higher percentage of participants with compulsory or no basic education, compared to the other clusters (17.1% vs. 13.7% in the *Metabolic* and 6.5% in the *Relatively healthy* clusters, *p* < .001). Whereas half of the *Relatively Healthy* cluster presented no chronic diseases, both *Metabolic* and *Psychiatric* clusters were entirely multimorbid (100% with two or more chronic diseases). With respect to pain dimensions, the mean of intensity, bothersomeness, and number of pain sites, as well the frequency of daily pain and severe pain showed a mild increasing trend from the *Relatively Healthy* cluster to the *Metabolic* cluster and further on to the *Psychiatric* cluster (*p* < .001). The neck, shoulder, and lower back were the most common pain locations within all clusters (Supplement Figure 1). Co-occurrence of two of these three locations showed also the highest prevalence rate within all clusters (Supplement Figure 2).

**Table 2.** Characteristics of the full sample and three clusters in terms of the background and pain variables (*n* = 4768).

	*Metabolic* (*n* = 502)	*Psychiatric* (*n* = 140)	*Relatively healthy* (*n* = 4126)	All (*n* = 4768)	*p* value
Sex, % (*n*)					
Women	49.0 (246)	50.7 (71)	56.3 (2322)	55.3 (2639)	.004
Men	51.0 (256)	49.3 (69)	43.7 (1804)	44.7 (2129)	
Educational level, % (*n*)					<.001
Compulsory or no basic education	13.7 (69)	17.1 (24)	6.5 (269)	7.6 (362)	
Secondary	66.7 (335)	61.4 (86)	64.7 (2668)	64.8 (3089)	
Tertiary	19.5 (98)	21.4 (30)	28.8 (1189)	27.6 (1317)	
Number of chronic diseases (range 0–15), % (*n*)					<.001
Zero	0 (0)	0 (0)	51.0 (2103)	44.1 (2103)	
One	0 (0)	0 (0)	35.8 (1479)	31.0 (1479)	
Two or more (multimorbidity)	100 (502)	100 (140)	13.2 (544)	24.9 (1186)	
Pain intensity, mean (SD)	4.7 (2.6)	5.3 (2.5)	4.0 (2.6)	4.1 (2.6)	<.001
Pain bothersomeness, mean (SD)	5.0 (3.0)	6.0 (2.8)	4.2 (2.9)	4.3 (2.9)	<.001
Number of pain sites, mean (SD)	4.3 (2.0)	5.0 (2.1)	3.8 (2.0)	3.9 (2.0)	<.001
Pain frequency over previous year*, % (*n*)					<.001
Daily	37.5 (188)	41.4 (58)	22.9 (944)	25.0 (1190)	
>30 d	35.9 (180)	35.7 (50)	37.3 (1539)	37.1 (1769)	
≤30 d	26.7 (134)	22.9 (32)	39.8 (1643)	37.9 (1175)	
Severe pain**					<.001
Yes	38.6 (194)	47.9 (67)	26.5 (1094)	28.4 (1355)	
No	61.4 (308)	52.1 (73)	73.5 (3032)	71.6 (3413)	

* The highest pain frequency of all pain locations.

** Pain that has lasted over 30 d within the previous year, is bothersome (numerical rating scale >5), and exists in two or more pain locations.

*p* value was calculated by chi-square test (categorical variables) and Kruskal–Wallis test (continuous variables).

### Clusters and MSK pain dimensions

MSK pain dimensions were compared across the three clusters using both the unadjusted and adjusted associations (Table 3). Compared to the *Relatively Healthy* cluster, the *Psychiatric* and *Metabolic* clusters had significantly higher pain intensity, bothersomeness, and the number of pain sites in the unadjusted models. The results remained fully equal after adjusting for sex and educational level (e.g. β and 95% CI for bothersomeness in the *Psychiatric* and *Metabolic* clusters: 1.8, 1.3–2.3; 0.8, 0.5–1.1, respectively). Similarly, participants in the *Psychiatric* and *Metabolic* clusters had over three-fold and over two-fold increase in adjusted odds of daily MSK pain, respectively, compared to the participants in the *Relatively Healthy* cluster (OR 3.04, 95% CI 1.96–4.74; 2.38, 1.87–3.02, respectively).

**Table 3.** Beta coefficients (β), odds ratios (ORs), and 95% confidence intervals (CI) for the unadjusted and adjusted associations between clusters and pain dimensions (*n* = 4768).

Unadjusted											
	Intensity		Bothersomeness		Number of pain sites		Frequency over previous year				
							Daily		>30 d		
Clusters	β	95% CI	β	95% CI	β	95% CI	OR	95% CI	OR	95% CI	≤30 d
*Metabolic*	**0.8**	(0.5–1.0)	**0.8**	(0.5–1.1)	**0.5**	(0.3–0.7)	**2.44**	(1.93–3.09)	**1.43**	(1.14–1.81)	Reference
*Psychiatric*	**1.3**	(0.9–1.8)	**1.8**	(1.3–2.3)	**1.1**	(0.8–1.5)	**3.16**	(2.03–4.89)	**1.67**	(1.07–2.61)	Reference
*Relatively healthy*	Reference		Reference		Reference		Reference		Reference		
Adjusted for sex and educational level											
*Metabolic*	**0.7**	(0.5–1.0)	**0.8**	(0.5–1.1)	**0.5**	(0.3–0.6)	**2.38**	(1.87–3.02)	**1.45**	(1.15–1.84)	Reference
*Psychiatric*	**1.3**	(0.9–1.7)	**1.8**	(1.3–2.3)	**1.1**	(0.8–1.4)	**3.04**	(1.96–4.74)	**1.69**	(1.08–2.66)	Reference
*Relatively healthy*	Reference		Reference		Reference		Reference		Reference		

β indicates the mean difference in the continuous outcome variable.

Bolded values are statistically significant at the 5% level.

### Clusters and severe MSK pain

Finally, cluster membership was examined as a determinant of severe MSK pain (defined as prolonged [over 30 d within the previous year], multisite (two or more pain sites), and bothersome [NRS >5] pain) (Table 4). The *Psychiatric* and *Metabolic* clusters had 155% and 75% higher odds of severe pain, compared to the *Relatively Healthy* cluster (adjusted OR 2.55, 1.81–3.59; 1.75, 1.44–2.13, respectively). The results were essentially identical in the unadjusted and adjusted models.

**Table 4.** Binomial logistic regression analysis for the associations between clusters and severe musculoskeletal pain, presented as odds ratios and 95% confidence intervals (*n* = 4768).

	Severe musculoskeletal pain 1. prolonged (lasted over 30 d within the previous year), 2. bothersome (numerical rating scale >5), and 3. multisite (two or more pain locations)	
	Unadjusted	Adjusted for sex and educational level
*Metabolic*	**1.75** (1.44–2.12)	**1.75** (1.44–2.13)
*Psychiatric*	**2.54** (1.81–3.57)	**2.55** (1.81–3.59)
*Relatively Healthy*	Reference	Reference

Bolded values are statistically significant at the 5% level.

## Discussion

We examined the distinct patterns of chronic disease accumulation in the general MSK pain population and studied whether they are differently associated with the severity of MSK pain, using pain frequency, intensity, bothersomeness, and the number of pain sites as outcomes. LCA identified three chronic disease clusters. A mental health disorder co-existed with a substance use disorder and a respiratory disease in the *Psychiatric* cluster, metabolic chronic diseases including obesity, hypertension, and diabetes accumulated in the *Metabolic* cluster, while the *Relatively Healthy* cluster represented participants with the lowest prevalence of chronic diseases. Both the *Psychiatric* and *Metabolic* clusters were associated with all the studied pain dimensions before and after adjustments for sex and educational level. Most importantly, participants in these clusters had 155% (CI: 81%–259%) and 75% (44%–113%) higher odds of severe MSK pain (defined as prolonged [over 30 days within the previous year], multisite (two or more pain sites), and bothersome [NRS >5] pain), respectively, compared to the individuals in the *Relatively Healthy* cluster.

Practically all individuals within the *Psychiatric* cluster had a mental health disorder. The appearance of the *Psychiatric* cluster was somewhat expected due to the substantial high prevalence of mental health disorders in pain populations [37–39] and their mutual relationship with MSK pain [37,40]. Substance use disorders have a strong correlation with mental health disorders [41,42], which was apparent in our data; that is, we found that the frequency of substance use disorder was markedly higher in the *Psychiatric* cluster than in the other clusters and in the full sample. The prevalence rates of respiratory diseases and stroke or other neurological diseases were 48.6% and 25.1%, respectively, within the *Psychiatric* cluster, indicating some co-existence of somatic health with mental health. Mental health disorders are reported to be at interplay not only with chronic obstructive pulmonary disease [43,44] but also with asthma [45,46]. Previously, a large LCA study in an osteoarthritic population, in which most participants were of working age, reported a corresponding cluster where depression occurred among 63% of the participants [20]. Asthma was most prevalent in that cluster as well. The mechanisms underlying the simultaneous occurrence of mental health disorders and MSK pain are not fully understood, but are likely represented in a multidimensional manner, with genetics [47,48], physiology/neurobiology (e.g. brain structure alterations [49] and neurotransmitters [37]), and shared lifestyle–psychosocial factors [50–53] playing a part.

Mental health disorders are known to be represented with a higher frequency along with ascending MSK pain severity [38] and to increase the odds of more intense and disabling pain phenotype [38,54,55]. The present study confirms these previous findings by showing that the *Psychiatric* cluster is associated with all pain dimensions, with mean intensity, bothersomeness, and the number of pain sites all being at least one NRS unit higher and the odds of daily pain three-fold higher when compared to the *Relatively healthy* cluster. Most importantly, the *Psychiatric* cluster had 155% higher odds of severe MSK pain, indicating that there are not only associations with single pain dimensions, but also with an adverse combination of them. Given the previously reported additive effects of mental health disorders and MSK pain e.g. on physical functioning, workability, and the level of primary health services use [38,56,57], the members of the *Psychiatric* cluster most likely require more comprehensive screening for by healthcare professionals and multimodal treatment approaches. Unfortunately, mental health disorders are often underdiagnosed in pain populations [37]. Based on our results, it should be kept in mind that individuals with MSK pain and a mental health disorder may also live with a concomitant substance use disorder or respiratory disease, which may also require treatment. Sleep deprivation, sociodemographics (e.g. work status), and psychological elements (e.g. pain catastrophizing and fear of pain) may have a role in the *Psychiatric* cluster–MSK pain associations [52,58–61].

According to our findings, metabolic diseases overlap in the MSK pain population as they appeared to form a group of their own. Obesity, hypertension, and diabetes often go hand in hand at the population level – particularly as obesity has a strong and negative influence on these cardiometabolic risk factors [62]. In accordance with the present results, a cardiometabolic cluster with the highest prevalence of cardiovascular risk factors and outcomes was also recorded among people living with osteoarthritis [20]. It is possible that chronic inflammatory state/other pathophysiological processes [62,63], mechanical stress (e.g. higher load or skeletal muscle strength deterioration related to obesity) [64], or congruent precursors, such as sedentary/inactive lifestyle [62,65] account for the detected accumulation of metabolic diseases in our MSK pain population. A part of MSK pain reports may also be related to complications of diabetes such as angio- or neuropathy. However, individuals who have diabetes, compared to those who have not, more often live with MSK pain [66], and diabetes was recorded ‘only’ among one-fourth of the *Metabolic* participants.

In addition to the *Psychiatric* cluster, the *Metabolic* cluster was associated with all pain dimensions and with 75% higher odds of severe pain, compared to the *Relatively Healthy* cluster. It is worth noting that although a higher percentage of individuals belonging to the *Metabolic* cluster than in the *Relatively Healthy* cluster (23.4% vs. 10.2%, respectively) also had a mental health disorder, the percentage was significantly lower than in the *Psychiatric* cluster (99.4%). Hence, the *Metabolic* cluster could be interpreted to present the worst pain with only a small difference in mental health. This is an important finding as it confronts the long-standing view that the worst mental health disorders accompany the worst pain [38,54,55]. Overall, our findings are consistent with prior studies showing that individuals with persistent MSK pain and metabolic syndrome (characterized by a combination of cardiovascular risk factors) experience higher pain intensity and disability, relative to counterparts with MSK pain only [67,68]. Yet the present study is among the first to show that accumulated metabolic diseases co-exist with the worst MSK pain. Even though there tends to exist a bidirectional association between the severity of MSK pain and metabolic diseases/risk factors [69], our present findings along with existing literature endorse the biopsychosocial nature of MSK pain [31,70] and reinforce the importance of paying attention not only to mental but also to metabolic health in people with MSK pain. Screening of metabolic health may be especially pertinent, considering the potential increased odds of premature death within the *Metabolic* cluster [20]. Metabolomics is an interesting area of research which could potentially explain the mechanisms driving the *Metabolic* cluster–severe MSK pain association [71,72], in addition to above discussed potential explanations.

Overall, the high prevalence of the *Relatively Healthy* cluster indicates that, fortunately, the majority of the individuals who report MSK pain are unlikely to have accumulated chronic diseases and their MSK pain tends to be less frequent, intense, and bothersome, and manifest in a more localized manner. Clusters defined by the low prevalence of chronic diseases have been associated with the lowest odds of disability, healthcare service use and mortality in other pain populations [19,20]. This current knowledge, supplemented by our findings, strongly indicates that a relatively healthy chronic disease profile is ostensibly favorable for better MSK health outcomes. Still, it is worth noting that one-fourth of the participants in the *Relatively Healthy* cluster had severe MSK pain. More detailed examination of the individuals who reported severe pain with a lack of chronic disease patterns may require further research.

A large study population of middle-aged Finns with data on a wide spectrum of pain dimensions is the principal strength of this study. With this data, we were able to study severe and thus potentially consequential MSK pain as an outcome and report the associations of distinct patterns of chronic disease accumulation with the severity of MSK pain among the first ones. Nevertheless, there are some limitations to this study. All data were ascertained through self-reports,which may have been influenced by subjective perceptions and social desirability. The cross-sectional study design did not allow us to make any conclusions about cause-and-effect relationships, thus either chronic diseases or MSK pain could have preceded the other. Still, our findings most likely underline the worrisome co-occurrence of mental and metabolic health disorders with severe MSK pain. Some sociodemographic differences have been reported between the NFBC1966 members who participated in the 46-year data collection and those who did not [25]. However, only minor discrepancies in the background variables were found between the sample of individuals with MSK pain and counterparts without [28]. Even though participants were asked about MSK pain in distinct MSK locations and those chronic diseases which primarily induce MSK pain were excluded, reported MSK pain may still be associated with some of the included chronic diseases or be neuropathic by its nature. Finally, we lacked data on the treatment balance of chronic diseases, e.g. whether or not a reported mental health disorder was stable and well-managed. This knowledge gap may have influenced the chronic disease patterns and their associations with the studied outcomes.

## Conclusions

The current observations suggest that individuals who have comorbid mental health disorders or accumulated metabolic diseases in addition to MSK pain experience more frequent, intense, bothersome, and multisite pain than those who are relatively healthy (i.e. less likely to have multiple chronic diseases in addition to MSK pain). Overall, the present study not only indicates that distinct patterns of chronic diseases can be identified in the general MSK pain population but also implies that the degree of MSK pain severity differs between these clusters. Importantly, it seems that not only mental health but also metabolic health interplay with MSK pain experiences, and often co-exist with severe MSK pain. As such, various chronic diseases that may co-occur with MSK pain, but do not appear to have a direct impact on MSK pain (e.g. respiratory disease and hypertension), may have a contributing role in MSK pain phenotype. In the clinical context, our findings indicate that clinicians should consider screening working-age MSK patients for mental and metabolic health entities, to characterize those who are more likely to experience the worst pain outcomes. Further research is needed to establish whether treatment and rehabilitation should be tailored in accordance with co-existing chronic diseases to achieve better pain outcomes. Furthermore, to establish the clinical significance, future studies should be conducted in other populations and in a longitudinal setting, and assessing e.g. health-related quality of life, healthcare service use, and mortality as outcomes. Similarly, the factors explaining chronic disease patterns warrant scientific attention.

## Supplementary Material

Supplemental Material

Supplemental Material

## Data Availability

NFBC data are available from the University of Oulu, Infrastructure for Population Studies. Permission to use the data can be applied for research purposes *via* the electronic material request portal. In the use of data, we follow the EU general data protection regulation (679/2016) and Finnish Data Protection Act. The use of personal data is based on the cohort participant’s written informed consent at his/her latest follow-up study, which may cause limitations to its use. Please, contact the NFBC project center (NFBCprojectcenter(at)oulu.fi) and visit the cohort website for more information.
