# Supplementary material for: Chronic disease clusters are associated with prolonged, bothersome, and multisite musculoskeletal pain: a population-based study on Northern Finns
Source: Ann Med. 2023 Feb 11;55(1):592–602. doi: 10.1080/07853890.2023.2177723 (PMC9930817; doi:10.1080/07853890.2023.2177723)
Supplement: Supplemental Material [file IANN_A_2177723_SM9337.docx]

**Supplement figure 2. Prevalence of concurrent musculoskeletal pain locations within the clusters (n=4,768).**
